# How could stress lead to major depressive disorder?

**DOI:** 10.1016/j.ibror.2018.04.001

**Published:** 2018-04-22

**Authors:** Gal Richter-Levin, Lin Xu

**Affiliations:** a Department of Neurobiology and Ethology, Department of Psychology, University of Haifa, Haifa 3498838, Israel; b Key Laboratory of Animal Models and Human Disease Mechanisms, Lab of Learning and Memory, Kunming Institute of Zoology, Chinese Academy of Sciences, Kunming 650223, China; c CAS Centre for Excellence in Brain Science and Intelligent Technology, Shanghai 200031, China; d Mental Health Institute, Second Xiangya Hospital of Central South University, Changsha 410011, China; e KIZ-SU Joint Laboratory of Animal Model and Drug Development, College of Pharmaceutical Sciences, Soochow University, Suzhou 215123, China

**Keywords:** Major depressive disorder, Stress, Memory

## Abstract

Stress is associated with major depressive disorder (MDD), but the underlying mechanism remains elusive. However, some experiences, referred to as stress, may actually lead to resilience. It is thus critical first to define what type of stress may lead to MDD. Long-term potentiation (LTP) and long-term depression (LTD) are both sensitive to stress, but particularly to inescapable and not escapable stress. Thus, these are the psychological aspects of stress which contribute to the development of MDD, but by which mechanisms remains still elusive. Interestingly, the same stress may facilitate LTD and impair LTP in the CA1 region. In addition, repeated efforts are often required for learning under neutral conditions but single- or few learning trials are sufficient for forming stress-related memories. If LTP is crucial for normal learning, a combination of limited LTP and facilitated LTD appears to have higher efficiency for storing stress-related memories. Chronic psychological stress may cause a hyper-link among stress-related memories across the spatiotemporal due to shared quality of inescapability, leading to automatically negative appraisal through memory generalization mechanisms in MDD patients when encountering new distinct events which are perceived to share such similarity with previous experiences.

## Psychological stress *vs.* stress

The idea that the effects of the physical properties of stress experience should be distinct from the psychological aspects of the experience has been put forward by the studies of the impact of uncontrollable versus controllable stress exposure (Seligman and Maier, 1967). The definitions of stress (Selye, 1936; Selye, 1955; Kim and Diamond, 2002) do not often emphasize the difference between physical and psychological aspects of stress despite the neurobiological effects produced by psychological and physical aspects of stress are likely to be much different. A definition of psychological stress should contain the features of inescapability and/or uncontrollability, as well as the diverse responses by individuals due to their own cognition and behavior pattern (such as mindset, learning and memory) etc. The impact of psychological stress on cognition is suggested to lead to rapid learning of adaptive or maladaptive coping strategies such as in the case of learned helplessness or immobility (Martin et al., 1967). These processes may lead to biased negative emotional processing on memory recall in MDD patients (Beck et al., 1961; Hollon and Kendall, 1980). If this view, which is supported by clinical observation about the causal relationship between stress and MDD (Caspi et al., 2003; Cohen et al., 2007; Mothersill and Donohoe, 2016; Bleys et al., 2018), is correct, the definition of psychological stress should include inescapability and/or uncontrollability. This differentiation between psychological and other forms of stress is further supported by the findings that acute inescapable but not escapable situation of the same stress facilitates LTD (Kim et al., 1996; Xu et al., 1997; Xu et al., 1998) and impairs LTP (Foy et al., 1987; Shors et al., 1990; Kim et al., 1996; Xu et al., 1997; Xu et al., 1998; Kim and Diamond, 2002) in the hippocampal CA1 region. These findings indicate that distinct neural mechanisms underlie psychological stress. While a role for stress-induced LTD in the development of MDD was suggested before (Manji et al., 2001; Duman and Aghajanian, 2012), the difference between psychological stress and stress, and the combination of impaired LTP and facilitated LTD related to MDD has been rarely discussed. In accordance with that, chronic stress models, which are known to induce MDD-related symptoms including the animals' learning that the situation is inescapable or uncontrollable. Furthermore, previous reports have demonstrated that after chronic stress exposure, hippocampal CA3 neurons exhibit loss of dendrite spines and atrophy of dendrites (Magarinos et al., 1996). Functional synaptic plasticity (e.g. LTP and LTD) is suggested to lead to structural synaptic plasticity such as synaptogenesis or ‘synaptopruning’ respectively (Collingridge et al., 2004), and chronic stress-induced structural changes are likely to attribute to LTD and synaptopruning.

In summary, it is suggested that under acute but mainly chronic psychological stress conditions, the alteration of synaptic plasticity and the formation of stress-related memories are both critical for the development of MDD as is reflected by particular biased learned cognition and behavioral patterns. This view suggests that in order to understand stress-induced MDD we also need to better understand whether chronic psychological stress induces LTD and synaptopruning, and how stress-related memories are stored.

Hans Selye coined the term ‘stress' to describe a non-specific response of an organism to stressors that have long been believed to be harmful for health (Selye, 1936; Selye, 1955). However, as was more recently realized, a stressor is not necessary bad or good, but good or bad is determined by the mindset of the individuals’ (Crum et al., 2013), suggesting that diverse responses of individuals to psychological stress depend on their formed cognition. Furthermore, the activation of the hypothalamic-pituitary-adrenal (HPA) axis by stress has long been considered to indicate and quantify stress (Kim and Diamond, 2002). However, particularly with respect to MDD, this is likely not an effective or sufficient measurement. A major risk factor for the development of MDD are stressful life events which are suggested to shape biased cognition and behavioral patterns that increase the likelihood to developing MDD in adulthood (Caspi et al., 2003; Cohen et al., 2007). It is likely not accurate to assess psychological stress in the past by measuring the levels of glucocorticoid hormones in the present. Indeed, differences in past events are likely to contribute to individual differences in responding to the same stress in adulthood (Myers and Brewin, 1994; Sandi and Richterlevin, 2009; Crum et al., 2013). The impact of the past on the present may be dependent on a particular retrieval mechanism, termed memory generalization, a phenomenon demonstrated in the famous story of ‘Little Albert’ who learned was trained to fear a white animal but then generalized his fear, responding to objects or animals with white color (Bower, 1981). Here, memory generalization may have been triggered by the similarity in characteristics related to inescapability and/or uncontrollability. In this way psychological stress and stress-related memories are likely to contribute to the development of MDD. Eventually, cognitive appraisal on the current stress situation (Folkman et al., 1986) and memory generalization (Bower, 1981) would lead to emotional changes that would contribute to the development of neuropsychiatric disorders including MDD.

## Evaluating previous effects of psychological stress

Cognitive appraisal has been examined in patients who suffer neuropsychiatric disorders especially MDD. For example, a neutral event or face is often recognized by MDD patients as “sadness”, a cognitive appraisal pattern that may be predominant by automatically negative thinking (Beck et al., 1961; Hollon and Kendall, 1980). This is highly consistent with a recent suggestion that a stress is bad because the individual believes the way (Crum et al., 2013), a mindset possibly resulted from cognitive appraisal that is made based on previous stress memories. It is therefore critical to understand how this could occur in MDD patients. There are three processes of a memory, encoding, storage, and retrieval (Atkinson and Shiffrin, 1968). The encoding-retrieval exactly matched conditions lead to specific recall but these of partial matched ones result in generalization recall, according to the theoretical hypothesis “Toward a universal law of generalization for psychological science” (Shepard, 1987). A recent report has demonstrated that rapid form of memory generalization is an active process to build up additional accesses to memory resources that allow fear to be transferred from one memory to another (Zhou et al., 2017). Clinical study has suggested that MDD patients exhibit over generalization of negative memories (Gotlib and Joormann, 2010). Therefore, according the rapid form of memory generalization, similar cues can retrieve negative emotion that links to old stress memories (Zhou et al., 2017; Silva, 2017), possibly leading to automatically negative thinking (Beck et al., 1961). Furthermore, psychological stress memories would have been accumulated to shape the cognitive appraisal pattern in individuals (Bower, 1981; Folkman et al., 1986). If the individuals encounter distinct new events but with similarity to the previous stressful events such as inescapability and/or uncontrollability, memory generalization mechanisms would transfer the negative emotion from the old stress memories to the present so as to manifest automatically negative thinking. Based on this speculation, a behavioral task carefully designed for testing generalization of the old stress memories would be very useful for evaluating the psychological effects of the past up to the present, to detect whether an individual is suffering or going to be suffering from MDD.

## Posttraumatic stress disorder (PTSD) *vs.* MDD

A general unsolved question is why a life-threatening event leads to PTSD or both PTSD and MDD in some, but only MDD in the others. Clinical study suggests that acute response to a trauma cannot distinguish PTSD from MDD, but MDD can be separated by chronic aftermath of the trauma (Odonnell et al., 2004). PTSD but not MDD is associated with certain physiological biomarkers such as hypertension (Kibler et al., 2009). Certain daily pattern of cortisol levels is also associated with PTSD, but not with MDD (Wichmann et al., 2017). It is interesting that memory retrieval is not affected in MDD patients but enhanced in PTSD patients after cortisol administration, suggesting that the most difference between MDD and PTSD may relate to memory retrieval mechanisms (Wingenfeld and Wolf, 2014). Furthermore, over generalization of stress memories in the retrieval is shared by MDD and PTSD (Gotlib and Joormann, 2010; Mahan and Ressler, 2012). Perhaps, accumulation of the life-threatening events may blunt the HPA axis activity, indirectly reflecting the cognitive appraisal for uncontrollability and/or inescapability so that these individuals may be more vulnerable in suffering MDD or the both (Sandi and Richterlevin, 2009). Moreover, not all stressful events are life-threatening or inescapable, but their effects would be accumulated through stress memories so as to forming inescapable concept and thus cognition appraisal pattern that determine the development of MDD. In short, PTSD may be triggered by a life-threatening event, but MDD may be triggered by such an event and along with accumulation of stress memories in the past.

## Suppression of stress-related memories may mediate the effects of fast acting antidepressant drugs

It is important to understand how psychological stress, following which LTP is impaired but LTD is facilitated, contributes to the formation of stress-related memories and how this may be related to MDD (Foy et al., 1987; Shors et al., 1990; Kim et al., 1996; Xu et al., 1997; Xu et al., 1998; Kim and Diamond, 2002). Towards that end it is important to verify whether related neurons in MDD patients or in animal models are more excited or inhibited. This may reflect on which therapy mechanism should be ideally developed for preventing or treating symptoms of MDD. A recent report demonstrates that PTSD-like or comorbidity with MDD-like animals show distinct patterns of c-fos activity in neurons of the limbic areas (Ritov et al., 2016), implicating that the states of PTSD and MDD are likely associated with different patterns of more excited neurons. These neurons may be involved in storing stress-related memories (Cai et al., 2016; Silva, 2017). Furthermore, in order to overcome the problem of delayed action of antidepressants, a cocktail of drugs, including antidepressants, anxiolytics, and antiepileptics are given to patients in order to achieve faster onset of antidepressant effects (Casacalenda and Boulenger, 1998; Ivkovic et al., 2009). These augmentation drugs are assumed to have inhibitory effects on neuronal activity, supporting the view that related neurons in MDD are likely more excited. In agreement with that, a low dose of ketamine, a well-known antagonist of NMDA receptors, can produce rapid (hours) and sustained (a week) antidepressant effects in MDD patients (Berman et al., 2000; Zarate et al., 2006) and in animal models (Autry et al., 2011; Koike et al., 2011; Jing et al., 2015; Zanos et al., 2016). However, recently a novel hypothesis was introduced to explain the rapid-onset antidepressant action of ketamine (Koike et al., 2011; Duman and Aghajanian, 2012; Kavalali and Monteggia, 2012; Gideons et al., 2014), suggesting that ketamine may have a fast action of enhancing AMPA receptor activity and synaptogenesis (Autry et al., 2011; Koike et al., 2011; Jing et al., 2015; Zanos et al., 2016). In light of these novel findings, can we really ignore the known effects of ketamine on NMDA receptors? (Miller et al., 2016). We argue that, assuming that ketamine or its metabolite can potentiate the activity of AMPA receptors only (Zanos et al., 2016), both effects may in fact complement each other. Given the well-known fact that excitation of neurons through AMPA receptors activity is the major prerequisite to open the NMDA receptor channels, the potentiation of AMPA receptors activity would unavoidably lead to more activity of NMDA receptors in the same neurons. While this may initially seem to be counterintuitive to the notion that the low-dose effect of ketamine, as an antagonist of NMDA receptors, inhibits the presumably over-excited neurons in MDD, both excitation of neurons through potentiation of AMPA receptor activity and partial blockade of NMDA receptors by low-dose of ketamine would together lead to LTD in glutamatergic synapses, as predicted by the sliding hypothesis of synaptic plasticity (known as BCM theory) (Bienenstock et al., 1982). If true, ketamine-induced LTD, such as found in the CA1 region of freely moving animals, would produce inhibitory effects on stress-related neurons. Such an effect can be expected to prevent retrieval of stress-related memories, as was demonstrated in a 2-trial forced swimming test (Duan et al., 2013). This idea is also consistent with the known effects on memory of the rapid-onset MDD treatment of electroconvulsive therapy (ECT). ECT leads to amnesia in patients and to post-ECT inhibition of neuronal activity in animals (Svensson et al., 2017). Another novel antidepressant treatment, deep brain stimulation (DBS) in the CA1 region may also disrupt the retrieval of stress-related memories, as is suggested by the finding that it reduces immobility in the second trial of forced swimming (Jing et al., 2015). Likewise, the recent evidence that optogenetic stimulation, aimed to induce LTD, can inactivate fear memory (Nabavi et al., 2014) is consistent with this notion, suggesting that retrieval of stress-related memories is suppressed by ketamine-induced LTD in MDD patients.

However, according to the BCM theory (Bienenstock et al., 1982), directly partial potentiation of NMDA receptors activity should also be able to induce LTD. It is thus reasonable to assume that such action can also produce rapid-onset antidepressant action. This mechanism is likely to be beneficial over the assumed dual-function of ketamine, because a side effect of psychosis can be avoided, and new memories may be formed to update the old stress-related memories, while the retrieval of old, biased memories may be inhibited since LTD mechanisms may be shared by psychological stress and the drug. Fortunately, we found a small molecule, termed orcinoside, derived from a traditional Chinese herb *rhizoma curculiginis*, which has been believed for over a thousand years to be able to enhance spiritual power and memory. Orcinoside targets the glycine site of NMDA receptors to partially promote its activity. Intriguingly, orcinoside was also found to weakly potentiate the AMPA receptor currents but only in some but not all neurons (Chen et al., 2007). The net effect of orcinoside is to induce a drug-dose dependent LTD in the CA1 regions of freely moving rats, which is very similar to the effects of ketamine (Duan et al., 2013). It is suggested that the antidepressant effect of orcinoside is attributable to decrease of neuronal excitation in certain neurons as a result of the combination of temporally and partially enhanced NMDA and AMPA receptors activity (Wang et al., 2015). If the stress-affected neurons are indeed more excited in MDD, we expect that these effects of orcinoside may produce rapid-onset antidepressant action in MDD patients. Accordingly, orcinoside is currently under the CFDA clinical trial phase IIb.

**Fig. 1:**
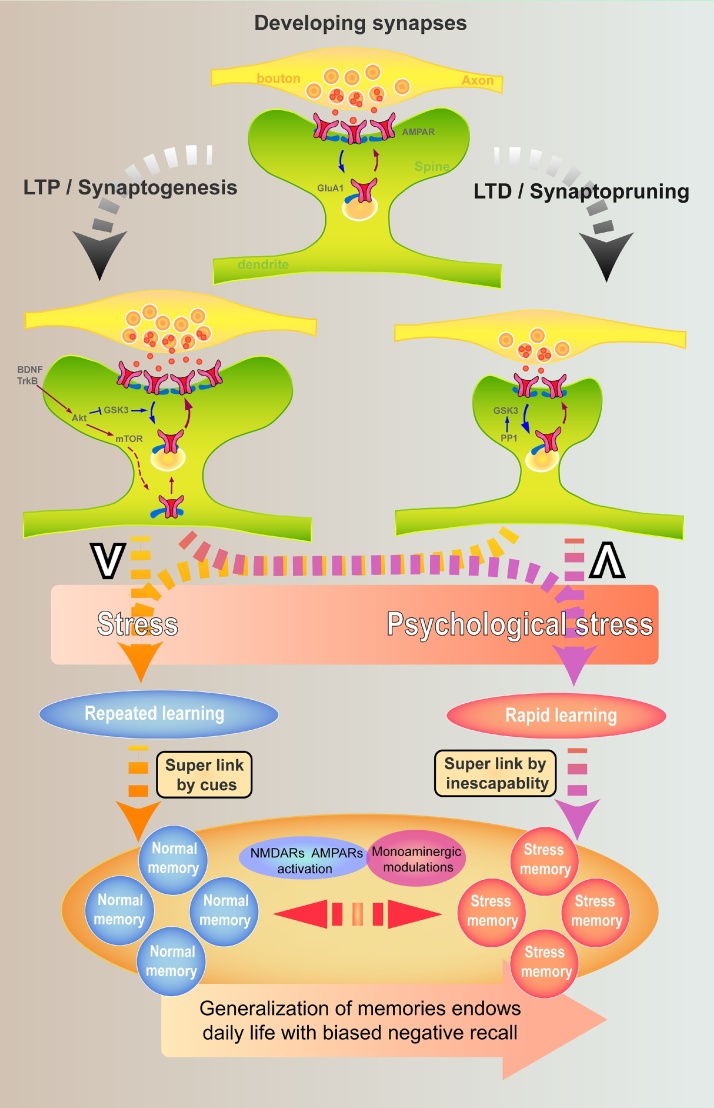
A schematic graph for how stress may lead to major depressive disorder (MDD). During the early development, the synapses are undergoing LTP/synaptogenesis and LTD/synaptopruning for building up neural circuits. Either LTP or LTD may endow repeated learning but a combination of LTP and LTD may enable rapid learning under psychological stress condition, the conserved mechanisms, which are critical for survival, sharing activations of NMDA receptor (NMDAR) and AMPA receptor (AMPAR) as well as modulations of monoaminergic systems. Biased negative emotion is individually different due to generalization of the stored stress memories. ∨ = LTP or LTD; ∧ = LTP and LTD.

How stressed neurons can store stress-related information is not yet understood because at least in the CA1 region psychological stress impairs LTP but facilitates LTD. One possibility is that psychological stress induces endogenous LTP in certain brain regions (Kim et al., 1996; Saal et al., 2003; Jing et al., 2015). However, this assumption is opposite to the current hypotheses of MDD, according to which stress is assumed to induce LTD, which leads to MDD while ketamine ameliorates symptoms by inducing chemical LTP (Manji et al., 2001; Duman and Aghajanian, 2012). It is important to note that either the mechanism of LTP or LTD is in fact not different from that of normal learning. The blockade of LTP by NMDA receptor antagonists impairs memory formation (Herron et al., 1986; Martin et al., 2000). The function of LTD is less clear, but it is suggested to be involved at least in certain types of learning in cerebellum (Linden, 1996) or regulating retrieval (Nabavi et al., 2014). Furthermore, the lack of LTD may lead to normal learning but impaired reversal learning (Li et al., 2015). What happens with regards to synaptic plasticity when encountering inescapable stress is not clear due to technique limitations. It is difficult to accurately assess which and how many synapses demonstrate LTP and/or LTD. Nevertheless, a reasonable assumption is that a combined LTP/LTD mechanism would have higher efficiency to store stress-related memories (Fig. 1). Thus, we assume that a combination of limited LTP and facilitated LTD in the synapses of the selected stress neurons by psychological stress would provide a more effective mechanism to store stress memories with little net changes in synaptic weight (Dudai, 1996; Xu et al., 1997).

## The possible translation to prevention and treatment of MDD

If psychological stress is associated with the development of MDD (Pittenger and Duman, 2008), antistress therapies would be the best strategy to prevent the development or relapse of MDD. Such practices may even help to cure the disease. Unfortunately, such therapies targeting psychological stress or the underlying mechanisms are as yet not effective enough (Hitchcock et al., 2017), although mindfulness approaches are considered helpful in reducing several biomarkers of physiological stress (Pascoe et al., 2017). It is interesting that capsaicin or its agonists can activate central TRPV1 channels and produce anti-stress effects on both synaptic plasticity and spatial memory under inescapable stress conditions (Li et al., 2008), implicating central TRPV1 channels as a likely target for developing novel drugs to prevent and treat MDD. However, treating MDD may turn out to be more difficult because of effects of previous exposures to psychological stress which lead to the accumulation of stress-related memories that bias cognitions and behavior. If stress-related memories are stored through LTD and synaptopruning accompanied by limited LTP and synaptogenesis, aiming to combined effects on both LTP and LTD may hold promise. Currently the best-known targets are to enhance synaptogenesis as ketamine does, but such treatment seem to affect only memory retrieval rather than the stress-related memories themselves so that the antidepressant action is short-lasting (about a week). Low dose of addictive drugs such as opiates, cannabis and ketamine are all able to induce the drug-dependent LTD in the CA1 region (Yang et al., 2004; Han et al., 2012; Duan et al., 2013), and their antidepressant action is rapid-onset but still short-lasting (several days).Therapies that will enable non-recovered drug-dose dependent LTD and synaptopruning the effects could be expected to be longer-lasting. Consistent with this idea, recent reports have demonstrated that optogenetic stimulation-induced LTD inactivates fear memory while stimulation-induced LTP activates it (Nabavi et al., 2014; Roy et al., 2016). A combination of drug-induced LTD with activated retrieval of the stress-related memories by drug-induced LTP will together allow the retrieved memories to be unstable thus eliminated more completely. In line with that direction it is possible to consider memory replacement, using new positive memories to counteract the impact of the old stress-related memories (Ramirez et al., 2015).

Monoaminergic systems-based drugs, which are still the drugs of choice for treating MDD, are possibly not the best choice for producing rapid-onset antidepressant action. We owe it to the patients to open our mind to consider other possibilities beyond the known antidepressants or the known mechanisms of antidepressant action, such as the active forgetting or rapid generalization mechanisms or the idea of updating old stress-related memories by novel positive memories (Shuai et al., 2010; Ramirez et al., 2015; Zhou et al., 2017). Deeper understanding of the neural mechanisms of the formation of stress-related memories will contribute to shaping such novel approaches.
